# National trends in repair for type B aortic dissection

**DOI:** 10.1002/clc.23672

**Published:** 2021-06-26

**Authors:** E. Hope Weissler, Oyomoare L. Osazuwa‐Peters, Melissa A. Greiner, G. Chad Hughes, Chandler A. Long, Sreekanth Vemulapalli, Manesh R. Patel, W. Schuyler Jones

**Affiliations:** ^1^ Division of Vascular and Endovascular Surgery Duke University School of Medicine Durham North Carolina USA; ^2^ Department of Population Health Sciences Duke University School of Medicine Durham North Carolina USA; ^3^ Division of Cardiovascular and Thoracic Surgery Duke University School of Medicine Durham North Carolina USA; ^4^ Division of Cardiology Duke University School of Medicine Durham North Carolina USA

**Keywords:** aortic surgery, claims analysis, TEVAR, type B aortic dissection

## Abstract

**Background:**

Thoracic endovascular aortic repair (TEVAR) first gained in popularity for repair of type B aortic dissections (TBADs) in the early 2000's. We aimed to describe patients undergoing open repair, TEVAR, and no repair and analyze factors associated with repair within 14 days of presentation in the contemporary era.

**Methods:**

We used the MarketScan database to find patients with TBAD between 2014 and 2017. To assess factors associated with early repair, univariable, and multivariable log‐binomial regression were used.

**Results:**

There were 2613 patients admitted with TBAD between 2014 and 2017 across the United States, of whom 38.4% underwent repair within 14 days of admission (25.3% open repair and 13.1% TEVAR). The incidence of repair within 14 days decreased over the study period (43% of the study cohort in 2014 to 26.4% in 2017) primarily due to a decrease in open repairs from 30.8% of patients in 2014 to 12.5% in 2017. In multivariable analysis, older age, Middle Atlantic location, diabetes mellitus, insulin use, antiplatelet use, and more recent year were associated with lower likelihood of early repair; male sex, peripheral vascular disease, and the presence of extremity ischemia, rupture, shock, and acidosis were associated with higher likelihood of repair.

**Conclusions:**

Overall, repair of TBAD within 14 days of presentation declined from 2014 to 2017, with a steady rate of TEVAR but declining rate of open repairs. Further investigation into provider‐ and hospital‐specific factors as they relate to likelihood of repair is needed.

## 1 INTRODUCTION

Historically, patients with uncomplicated TBAD (without rupture or malperfusion) have been treated medically while patients with complicated TBAD were repaired with open surgery or thoracic endovascular aortic repair (TEVAR). Academic interest in the effectiveness of TEVAR among TBAD patients without rupture or malperfusion has grown (as shown by the INSTEAD and ADSORB trials among many other observational reports),^1^, ^2^ and analyses of national patterns of TBAD repair in the early 2000s–2010s documented increases in the frequency of repair and proportion of TEVARs over time.^3^, ^4^, ^5^, ^6^, ^7^, ^8^ However, these analyses spanned the period of early off‐label TEVAR for TBAD beginning in 2000 through FDA approval of a dissection‐specific thoracic stent graft in 2013, and repair patterns in modern clinical practice following these paradigm‐shifting developments have not been characterized. In addition to understanding the overall frequency and types of repairs, detailing features of patients undergoing repairs and specific types of repairs offers a way to study variability in TBAD clinical care and outcomes nationally.

We aimed to use the MarketScan database, which includes claims from millions of Americans with private insurance without age restriction, to analyze patients presenting with TBAD between 2014 and 2017. We intended to describe trends in repair frequency and type over the study period; identify differences between patients undergoing open repair, TEVAR, or no repair within 14 days of hospital admission; and to elucidate factors associated with repair versus no repair.

## 2 METHODS

### 2.1 Data source

We used the MarketScan commercial claims database (IBM Research, Yorktown Heights, NY), which is a proprietary dataset consisting of inpatient admissions and services claims, outpatient services and pharmaceutical claims, facility headers, and enrollment details for over 50 million patients with private insurance in the United States. This study was determined exempt by our local institutional review board and no patient consent was required (Pro00104988). Because this study used de‐identified patient data, no informed consent was obtained.

### 2.2 Patient population

The cohort consisted of adult (≥18 years) patients with index inpatient admissions for TBAD between January 1, 2014 and December 31, 2017. Inclusion criteria included an inpatient hospitalization with ICD‐9‐CM (January 1, 2014–September 30, 2015) or ICD‐10‐CM (October 1, 2015–December 31, 2017) codes for thoracic or thoracoabdominal aortic dissection in any diagnosis code position: ICD‐9‐CM (441.01, 441.03), ICD‐10‐CM (I71.01, I71.03). For patients with multiple such admissions during the study period, the earliest admission was used.

**FIGURE 1:**
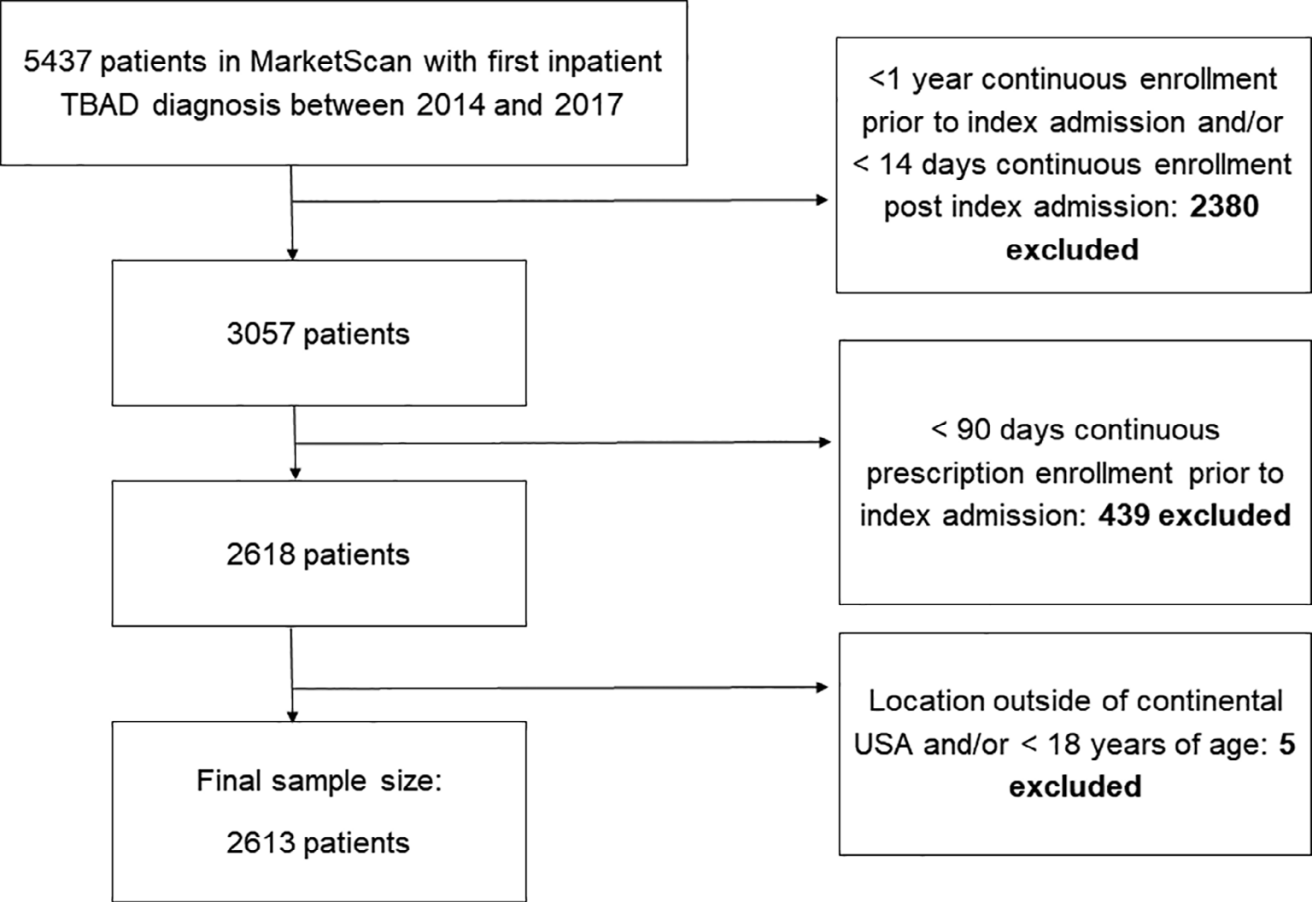
CONSORT diagram. Patients were excluded for lack of coverage prior to index admission in order to ascertain comorbidities and prescription medication use

Exclusion criteria included an inpatient admission with a thoracic or thoracoabdominal aortic dissection code between January 1, 2013 and December 31, 2013. Due to data availability, patients were not able to be excluded on the basis of dissection codes prior to 2013; therefore, some patients in the cohort may have been hospitalized due to progression or complications of chronic dissections. Patients with type A aortic dissections were removed by excluding patients with concomitant procedure codes for cardioplegia, cardiopulmonary bypass, valve repair, or operations on the vessels of the heart, following previously‐described coding strategies (Appendix A).^9^ Patients without 1 year of continuous insurance enrollment (for comorbidity ascertainment) and 90 days of continuous prescription coverage (for medication usage ascertainment) prior to their index admission, as well as 14 days of continuous insurance enrollment post index admission (for repair ascertainment) were excluded (Figure 1). Though mortality is not reported in MarketScan, removal of enrollment ID from the dataset may indicate death, loss of health insurance, or discontinuation of an insurance's participation in the MarketScan database.

### 2.3 Outcomes and covariates

The primary outcome was the presence and type of repair within 14 days of index admission. This time window was chosen in accordance with the 2020 Society for Vascular Surgery and Society of Thoracic Surgeons reporting standards for TBAD.^10^ Open surgical repair and TEVAR were defined by ICD‐9‐PCS, ICD‐10‐PCS, and CPT codes as given in Table 1.

**TABLE 1:** Procedure code definitions for open repair and TEVAR

	ICD‐9 PCS	ICD‐10 PCS	CPT
Open repair	38.34, 38.45, 38.65, 39.31, 39.57	02QW0ZZ, 02RW07Z, 02RW08Z, 02RW0JZ, 02RW0KZ 02UW07Z, 02UW08Z, 02UW0JZ, 02UW0KZ 02BW0ZZ	34830, 33875, 33877, 33870, 33335, 33330, 34839
TEVAR	39.73, 39.79	02UW3JZ, 02QW3ZZ	33880, 33881, 33883, 33884, 33886, 0254 T, 0255 T, 0078 T, 0079 T, 0080 T, 0081 T, 3800, 34800, 34802, 34803, 34804, 34805, 34808, 34812, 34813, 34820, 34825, 34826, 34833, 34834, 34841, 34842, 34843, 34844, 34845, 34846, 34847, 34848, 34900, 35472

Abbreviations: CPT, Current Procedural Terminology; ICD, International Classification of Diseases; PCS: Procedure Coding System.

Other variables of interest included demographics, insurance plan type (divided into those with and without primary care clinician assignments), and comorbidities. Patient race and ethnicity are not available in MarketScan. Comorbidities were ascertained using previously validated coding algorithms in the year prior to, and exclusive of, the index admission date.^11^, ^12^ Medication use was assessed using filled medication prescriptions from 90 days prior to, and not inclusive of, the index admission date. Medication types included antihypertensives, anticoagulants, antiplatelets, statins and other lipid lowering agents, and insulin (Appendix B).

Complications during index admission were ascertained using ICD‐9‐CM and ICD‐10‐CM codes and included aortic rupture, bowel ischemia, extremity ischemia, acidosis, shock, renal failure, paraplegia, and stroke (Appendix C). Because the diagnosis codes used to determine the presence of complications were captured from the billing claim filed at the time of discharge, it was not possible to determine whether they were present at the time of admission or developed following repair. Therefore, it was not possible to divide the cohort into complicated versus uncomplicated dissections. Because of the lack of mortality data in MarketScan, we were not able to report mortality during or following admission. We were also unable to report morbidity following the index admission, because mortality is a significant competing risk for morbidity in this patient cohort.

### 2.4 Statistical analysis

Baseline patient characteristics were described overall and by groups of open repair within 14 days of admission, TEVAR within 14 days of admission, and no early repair. Differences in baseline characteristics between the groups were tested using Pearson's chi‐square tests for categorical variables and the Wilcoxon rank‐sum tests for continuous variables. In order to assess factors associated with early repair, patients undergoing open repair and TEVAR were combined into a single group and univariable and multivariable log‐binomial regression were used. Log‐binomial regression was chosen because the early repair rate was >10%.^13^ For ordinal and nominal variables with multiple levels, the level or category with the highest frequency of patients was used as the reference. We used a 2‐tailed *α* = .05 to establish statistical significance and reported 95% confidence intervals. Statistics were carried out using SAS (Cary, NC).

## 3 RESULTS

### 3.1 Cohort description, presence/absence of repair type

There were 2613 patients admitted with TBAD between 2014 and 2017 who fulfilled the inclusion and exclusion criteria. Overall, 1002 patients underwent repair within 14 days of admission (341 (13.1%) TEVAR, 661 (25.3%) open) and 1611 (61.7%) patients did not undergo repair within 14 days of admission (Table 2). Patients undergoing repair within 14 days of admission were younger (open: median age 59.0, IQR 51.0–67.0; TEVAR: median age 60.0, IQR 51.0–72.0; none: median age 63.0, IQR 54.0–77.0, *p <* .001). Patients undergoing open repair were more frequently male (open: 71.3%, TEVAR: 61.9%, none: 58.0%, *p <* .001).

**TABLE 2:** Cohort demographics and comorbidities

	Overall = 2613 N (%)	Open repair = 661 N (%)	TEVAR = 341 N (%)	No early repair = 1611 N (%)	*p*‐value
Demographics					
Age, years: median (IQR)	62.0 (53.0, 74.0)	59.0 (51.0, 67.0)	60.0 (51.0, 72.0)	63.0 (54.0, 77.0)	<.001
Age group					<.001
≤40	160 (6.1)	38 (5.7)	33 (9.7)	89 (5.5)	
40–49	294 (11.3)	103 (15.6)	43 (12.6)	148 (9.2)	
50–59	650 (24.9)	197 (29.8)	90 (26.4)	363 (22.5)	
60–69	646 (24.7)	186 (28.1)	71 (20.8)	389 (24.1)	
70–79	451 (17.3)	97 (14.7)	64 (18.8)	290 (18.0)	
80–89	344 (13.2)	39 (5.9)	37 (10.9)	268 (16.6)	
90+	68 (2.6)	1	3	64 (4.0)	
Male	1617 (61.9)	471 (71.3)	211 (61.9)	935 (58.0)	<.001
Health plan type					.150
Primary care provider (PCP)	2152 (82.4)	539 (81.5)	288 (84.5)	1325 (82.2)	
No PCP	429 (16.4)	116 (17.5)	52 (15.2)	261 (16.2)	
Unknown	32 (1.2)	6	1	25 (1.6)	
Year of index admission					<.001
2014	811 (31.0)	250 (37.8)	99 (29.0)	462 (28.7)	
2015	656 (25.1)	184 (27.8)	95 (27.9)	377 (23.4)	
2016	658 (25.2)	166 (25.1)	79 (23.2)	413 (25.6)	
2017	488 (18.7)	61 (9.2)	68 (19.9)	359 (22.3)	
Admission on week day	1996 (76.4)	518 (78.4)	271 (79.5)	1207 (74.9)	.025
US census divisions					.001
New England	96 (3.7)	21 (3.2)	11 (3.2)	64 (4.0)	
Middle Atlantic	436 (16.7)	87 (13.2)	46 (13.5)	303 (18.8)	
East North Central	662 (25.3)	176 (26.6)	83 (24.3)	403 (25.0)	
West North Central	122 (4.7)	32 (4.8)	16 (4.7)	74 (4.6)	
South Atlantic	553 (21.2)	121 (18.3)	79 (23.2)	353 (21.9)	
East South Central	145 (5.5)	38 (5.7)	25 (7.3)	82 (5.1)	
West South Central	242 (9.3)	68 (10.3)	32 (9.4)	142 (8.8)	
Mountain	108 (4.1)	38 (5.7)	10	60 (3.7)	
Pacific	207 (7.9)	69 (10.4)	28 (8.2)	110 (6.8)	
Unknown	42 (1.6)	11 (1.7)	11 (3.2)	20 (1.2)	
Comorbidities					
Hypertension	1768 (67.7)	415 (62.8)	222 (65.1)	1131 (70.2)	<.001
Diabetes mellitus	437 (16.7)	69 (10.4)	40 (11.7)	328 (20.4)	<.001
Ischemic heart disease	757 (29.0)	150 (22.7)	87 (25.5)	520 (32.3)	<.001
Congestive Heart failure	385 (14.7)	67 (10.1)	44 (12.9)	274 (17.0)	<.001
Cerebrovascular disease	392 (15.0)	75 (11.3)	46 (13.5)	271 (16.8)	.001
History of stroke	217 (8.3)	39 (5.9)	21 (6.2)	157 (9.7)	<.001
Chronic obstructive pulmonary disease	572 (21.9)	110 (16.6)	62 (18.2)	400 (24.8)	<.001
Cardiac arrhythmias	718 (27.5)	143 (21.6)	83 (24.3)	492 (30.5)	<.001
Peripheral vascular disease	1212 (46.4)	294 (44.5)	177 (51.9)	741 (46.0)	.615
Renal disease	314 (12.0)	48 (7.3)	44 (12.9)	222 (13.8)	<.001
Valvular heart disease	668 (25.6)	172 (26.0)	74 (21.7)	422 (26.2)	.349
Medication in prior 90 days					
Anti‐hypertensive medications	1490 (57.0)	350 (53.0)	173 (50.7)	967 (60.0)	<.001
Anticoagulant medications	312 (11.9)	65 (9.8)	32 (9.4)	215 (13.3)	.005
Antiplatelet medications	128 (4.9)	12 (1.8)	12 (3.5)	104 (6.5)	<.001
Insulin	58 (2.2)	6	4	48 (3.0)	<.001
Statins and other lipid lowering agents	818 (31.3)	179 (27.1)	96 (28.2)	543 (33.7)	<.001
Complications in index presentation					
Rupture	170 (6.5)	50 (7.6)	43 (12.6)	77 (4.8)	<.001
Bowel ischemia	34 (1.3)	6	6	22 (1.4)	.713
Extremity ischemia	112 (4.3)	32 (4.8)	27 (7.9)	53 (3.3)	.001
Acidosis	186 (7.1)	78 (11.8)	20 (5.9)	88 (5.5)	<.001
Shock	167 (6.4)	78 (11.8)	16 (4.7)	73 (4.5)	<.001
Renal failure	572 (21.9)	170 (25.7)	64 (18.8)	338 (21.0)	.154
Paraplegia	87 (3.3)	34 (5.1)	13 (3.8)	40 (2.5)	.002
Stroke	272 (10.4)	99 (15.0)	25 (7.3)	148 (9.2)	.010

Hypertension was the most frequent comorbidity overall, present in 62.8% of patients undergoing open repair, 65.1% of patients undergoing TEVAR, and 70.2% of patients not undergoing repair (*p <* .001). Patients undergoing repair within 14 days of admission, whether open or TEVAR, generally had lower rates of comorbidities than patients who did not have repair. However, patients undergoing TEVAR were numerically, but insignificantly, more likely to have peripheral vascular disease (open: 44.5%, TEVAR: 51.9%, none: 46.0%, *p =* .615).

Antihypertensive medications were the most commonly taken medications in the 90 days prior to index presentation (open: 53.0%, TEVAR: 50.7% none: 60.0%, *p <* .001). Statin and other lipid lowering agents were the second most commonly taken medications (open: 27.1%, TEVAR: 28.2%, none: 33.7%, *p <* .001). Few patients took anticoagulant medications (11.9% overall) or antiplatelet medications (4.9% overall) in the 90 days before TBAD admission.

Aortic rupture was present in 6.5% of the overall cohort (open: 7.6%, TEVAR: 12.6%, none: 4.8%, *p <* .001). Extremity ischemia was present in 4.3% of the overall cohort (open: 4.8%, TEVAR 7.9%, none: 3.3%, *p =* .001), and bowel ischemia in 1.3% (*p >* .05). Paraplegia and stroke were also more common among patients undergoing repair within 14 days of admission (paraplegia – open: 5.1%, TEVAR 3.8%, none: 2.5%, *p =* .002; stroke – open 15.0%, TEVAR: 7.3%, none: 9.2%, *p =* .010). Acidosis and shock were significantly more common among patients undergoing open repair (open: 11.8% and 11.8%, respectively; TEVAR: 5.9% and 4.7%, respectively; none: 5.5% and 4.5%, respectively; *p <* .001 for both). There were no significant differences in the frequency of renal failure between groups, though the overall frequency was high (21.9%). There were 462 patients who underwent repair within 14 days of admission who did not have any codes for complications during the index admission (46.1% of patients undergoing repair within 14 days).

### 3.2 Temporal trends in the cohort and early repair

**FIGURE 2:**
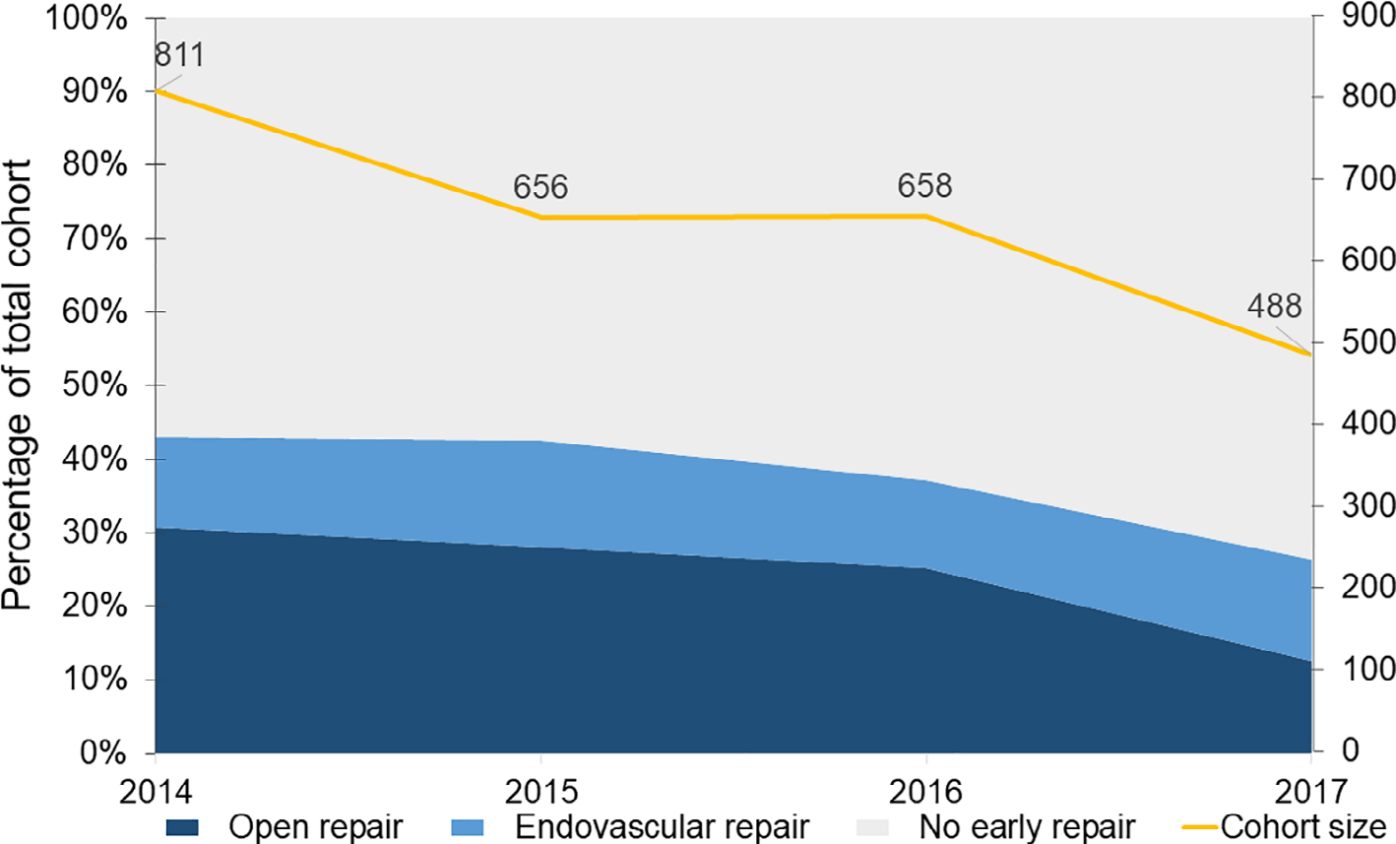
Change in overall cohort size and relative proportion of repair over time. The overall cohort size and proportion of open repair decreased over time while the proportion of TEVAR remained the same

When examining temporal trends, the overall annual MarketScan record counts decreased over time, from 47.85 million in 2014 to 26.15 million in 2017, due to changes in contributing insurance carriers. Similarly, the number of patients meeting the cohort inclusion criteria decreased from 811 in 2014 (0.0016% of enrolled patients) to 488 in 2017 (0.0019% of enrolled patients). The percentage of repair within 14 days of admission in general decreased over time (Figure 2), from 43% of the study cohort in 2014 to 26.4% in 2017. This was driven by a decrease in frequency of open repair from 30.8% of patients in 2014 to 12.5% in 2017. The percentage of TEVAR in the overall cohort remained relatively stable over time (12.2% of patients in 2014 versus 13.9% of patients in 2017) but the relative proportion of TEVAR versus open repair increased over time from 28.4% of repairs in 2014 to 52.7% of repairs in 2017. There were no significant differences in age, comorbidities, medication use, or complications coded during index admission from year to year (all *p >* .05, Table 3).

**TABLE 3:** Temporal trends in baseline characteristics and repair

	2014 N (% TBAD cohort)	2015 N (% TBAD cohort)	2016 N (% TBAD cohort)	2017 N (% TBAD cohort)	*p* value
Enrollment summary (millions)	47.85	29.62	28.72	26.15	
TBAD index admissions	811	656	658	488	
Demographics					
Age, years: median (IQR)	62.0 (53.0, 75.0)	61.0 (52.0, 74.0)	63.0 (55.0, 74.0)	62.0 (53.0, 72.5)	.154
Age group					.050
≤40	41 (5.1)	55 (8.4)	40 (6.1)	24 (4.9)	
40–49	98 (12.1)	73 (11.1)	59 (9.0)	64 (13.1)	
50–59	204 (25.2)	166 (25.3)	168 (25.5)	112 (23.0)	
60–69	183 (22.6)	148 (22.6)	176 (26.7)	139 (28.5)	
70–79	156 (19.2)	112 (17.1)	106 (16.1)	77 (15.8)	
80–89	114 (14.1)	86 (13.1)	86 (13.1)	58 (11.9)	
90+	15 (1.8)	16 (2.4)	23 (3.5)	14 (2.9)	
Male	510 (62.9)	377 (57.5)	411 (62.5)	319 (65.4)	.039
Health plan type					.005
Primary care provider (PCP)	693 (85.5)	541 (82.5)	537 (81.6)	381 (78.1)	.008
No PCP	111 (13.7)	109 (16.6)	115 (17.5)	94 (19.3)	.049
Unknown	7	6	6	13 (2.7)	.016
Admission on week day	617 (76.1)	505 (77.0)	502 (76.3)	372 (76.2)	.981
US census divisions					<.001
New England	31 (3.8)	21 (3.2)	22 (3.3)	22 (4.5)	.653
Middle Atlantic	131 (16.2)	116 (17.7)	104 (15.8)	85 (17.4)	.755
East North Central	197 (24.3)	178 (27.1)	180 (27.4)	107 (21.9)	.111
West North Central	39 (4.8)	34 (5.2)	35 (5.3)	14 (2.9)	.203
South Atlantic	168 (20.7)	135 (20.6)	123 (18.7)	127 (26.0)	.023
East South Central	55 (6.8)	27 (4.1)	34 (5.2)	29 (5.9)	.154
West South Central	50 (6.2)	72 (11.0)	69 (10.5)	51 (10.5)	.004
Mountain	39 (4.8)	21 (3.2)	36 (5.5)	12 (2.5)	.032
Pacific	69 (8.5)	48 (7.3)	52 (7.9)	38 (7.8)	.868
Unknown	32 (3.9)	4	3	3	<.001
Comorbidities					
Hypertension	552 (68.1)	439 (66.9)	442 (67.2)	335 (68.6)	.916
Diabetes mellitus	136 (16.8)	102 (15.5)	112 (17.0)	87 (17.8)	.772
Ischemic heart disease	233 (28.7)	192 (29.3)	190 (28.9)	142 (29.1)	.996
Congestive heart failure	125 (15.4)	104 (15.9)	95 (14.4)	61 (12.5)	.402
Cerebrovascular disease	119 (14.7)	90 (13.7)	103 (15.7)	80 (16.4)	.599
History of stroke	63 (7.8)	47 (7.2)	60 (9.1)	47 (9.6)	.373
Chronic obstructive pulmonary disease	189 (23.3)	133 (20.3)	149 (22.6)	101 (20.7)	.462
Cardiac arrhythmias	224 (27.6)	168 (25.6)	189 (28.7)	137 (28.1)	.625
Peripheral vascular disease	383 (47.2)	296 (45.1)	302 (45.9)	231 (47.3)	.827
Renal disease	88 (10.9)	69 (10.5)	92 (14.0)	65 (13.3)	.132
Valvular heart disease	213 (26.3)	162 (24.7)	167 (25.4)	126 (25.8)	.919
Medication in prior 90 days					
Anti‐hypertensive medications	482 (59.4)	366 (55.8)	367 (55.8)	275 (56.4)	.418
Anticoagulant medications	94 (11.6)	70 (10.7)	80 (12.2)	68 (13.9)	.396
Antiplatelet medications	39 (4.8)	34 (5.2)	31 (4.7)	24 (4.9)	.981
Insulin	16 (2.0)	18 (2.7)	14 (2.1)	10	.765
Statins and other antilipidemic medications	263 (32.4)	196 (29.9)	200 (30.4)	159 (32.6)	.632
Complications in index presentation					
Rupture	43 (5.3)	48 (7.3)	48 (7.3)	31 (6.4)	.343
Bowel ischemia	10	11 (1.7)	11 (1.7)	2	.215
Extremity ischemia	24 (3.0)	26 (4.0)	35 (5.3)	27 (5.5)	.066
Acidosis	53 (6.5)	42 (6.4)	54 (8.2)	37 (7.6)	.520
Shock	50 (6.2)	42 (6.4)	41 (6.2)	34 (7.0)	.947
Renal failure	180 (22.2)	149 (22.7)	147 (22.3)	96 (19.7)	.618
Paraplegia	23 (2.8)	28 (4.3)	19 (2.9)	17 (3.5)	.416
Stroke	76 (9.4)	78 (11.9)	64 (9.7)	54 (11.1)	.386
Frequency of repair and type					
Open repair	250 (30.8)	184 (28.0)	166 (25.2)	61 (12.5)	<.001
Endovascular repair	99 (12.2)	95 (14.5)	79 (12.0)	68 (13.9)	
No early repair	462 (57.0)	377 (57.5)	413 (62.8)	359 (73.6)	

### 3.3 Factors associated with early repair versus no repair

Factors associated with early repair on univariable analysis can be seen in Table 4. After adjustment for baseline characteristics, age 80–89 (aRR 0.62, 95% CI 0.49–0.77) and 90+ (aRR 0.16, 95% CI 0.06–0.41) remained associated with lower likelihood of repair within 14 days of admission (Table 4). Male sex remained associated with higher likelihood of repair (aRR 1.18, 95% CI 1.07–1.31) as did weekday admission (aRR 1.15, 95% CI 1.03–1.29). The Middle Atlantic region remained associated with lower likelihood of repair within 14 days (aRR 0.79, 95% CI 0.68–0.93). Diabetes mellitus was the only comorbidity associated with lower likelihood of repair after adjustment (aRR 0.75, 95% CI 0.63–0.89), but peripheral vascular disease was associated with repair after adjustment (aRR 1.12, 95% CI 1.01–1.24). Antiplatelet use was the only medication that remained associated with lower likelihood of repair after adjustment for other patient and index presentation factors (aRR 0.65, 95% CI 0.45–0.94). Year of index admission continued to be associated with lower likelihood of repair (2016: RR 0.89, 95% CI 0.78–1.00; 2017: RR 0.61, 95% CI 0.52–0.72).

**TABLE 4:** Univariable and multivariate analysis of factors associated with early repair

	Unadjusted risk ratio (95% CI)	*p* value	Adjusted risk ratio (95% CI)	*p* value
Demographics				
Age group				
≤40	0.99 (0.81–1.21)	.92	0.97 (0.80–1.17)	.74
40–49	1.12 (0.97–1.30)	.11	1.10 (0.96–1.27)	.16
50–59	1.00 (Reference)		1.00 (Reference)	
60–69	0.89 (0.78–1.02)	.08	0.94 (0.83–1.07)	.36
70–79	0.81 (0.69–0.95)	.008	0.87 (0.75–1.01)	.07
80–89	0.53 (0.42–0.65)	<.001	0.62 (0.49–0.77)	<.001
90+	0.11 (0.04–0.33)	<.001	0.16 (0.06–0.41)	<.001
Male	1.31 (1.18–1.46)	<.001	1.18 (1.07–1.31)	.001
Health plan type				
Primary care provider (PCP)	1.00 (Reference)		1.00 (Reference)	
No PCP	1.04 (0.91–1.18)	.59	1.01 (0.89–1.14)	.86
Unknown	0.53 (0.26–1.09)	.08	0.64 (0.33–1.22)	.17
Admission on week day	1.15 (1.01–1.29)	.03	1.15 (1.03–1.29)	.01
Year of index admission				
2014	1.00 (Reference)		1.00 (Reference)	
2015	1.00 (0.89–1.13)	.99	1.03 (0.92–1.16)	.56
2016	0.87 (0.77–0.99)	.04	0.89 (0.78–1.00)	.05
2017	0.63 (0.53–0.76)	<.001	0.61 (0.52–0.72)	<.001
US census divisions				
New England	0.82 (0.61–1.12)	.21	0.83 (0.62–1.11)	.22
Middle Atlantic	0.76 (0.63–0.90)	.002	0.79 (0.68–0.93)	.005
East North Central	1.00 (Reference)		1.00 (Reference)	
West North Central	1.01 (0.79–1.28)	.95	0.91 (0.72–1.13)	.39
South Atlantic	0.93 (0.81–1.08)	.36	0.94 (0.81–1.08)	.35
East South Central	1.08 (0.88–1.34)	.46	1.02 (0.84–1.24)	.83
West South Central	1.04 (0.86–1.24)	.7	1.03 (0.87–1.22)	.74
Mountain	1.10 (0.87–1.39)	.44	1.01 (0.82–1.24)	.93
Pacific	1.18 (0.99–1.40)	.07	1.10 (0.93–1.30)	.27
Unknown	1.31 (0.97–1.77)	.08	1.07 (0.81–1.42)	.62
Comorbidities and medications				
Hypertension	0.83 (0.76–0.92)	<.001	1.06 (0.94–1.18)	.33
Diabetes mellitus	0.61 (0.51–0.72)	<.001	0.75 (0.63–0.89)	.001
Ischemic heart disease	0.76 (0.67–0.86)	<.001	0.91 (0.81–1.04)	.16
Congestive heart failure	0.72 (0.61–0.85)	<.001	0.98 (0.83–1.16)	.84
Cerebrovascular disease	0.78 (0.67–0.91)	.002	1.10 (0.90–1.34)	.36
History of stroke	0.70 (0.56–0.88)	.002	0.82 (0.63–1.08)	.16
Chronic obstructive pulmonary disease	0.74 (0.65–0.85)	<.001	0.88 (0.77–1.01)	.08
Cardiac arrhythmias	0.77 (0.68–0.87)	<.001	0.94 (0.82–1.08)	.38
Peripheral vascular disease	1.03 (0.93–1.13)	.61	1.12 (1.01–1.24)	.03
Renal disease	0.74 (0.62–0.89)	.001	0.91 (0.76–1.09)	.3
Valvular heart disease	0.95 (0.85–1.06)	.35	1.05 (0.93–1.19)	.44
Anti‐hypertensive medications	0.82 (0.75–0.91)	<.001	0.95 (0.85–1.06)	.35
Anticoagulant medications	0.79 (0.66–0.94)	.008	0.95 (0.79–1.14)	.57
Antiplatelet medications	0.48 (0.33–0.69)	<.001	0.65 (0.45–0.94)	.02
Statins and other antilipidemic medications	0.83 (0.74–0.93)	.001	1.04 (0.93–1.17)	.47
Insulin	0.44 (0.25–0.78)	.005	0.65 (0.36–1.16)	.14
Complications in index admission				
Rupture	1.47 (1.27–1.70)	<.001	1.32 (1.14–1.51)	<.001
Bowel ischemia	0.92 (0.58–1.45)	.72	0.70 (0.45–1.08)	.1
Extremity ischemia	1.40 (1.16–1.68)	<.001	1.26 (1.05–1.50)	.01
Acidosis	1.41 (1.22–1.64)	<.001	1.29 (1.11–1.49)	<.001
Shock	1.52 (1.31–1.75)	<.001	1.45 (1.26–1.68)	<.001
Renal failure	1.09 (0.97–1.22)	.15	0.99 (0.88–1.10)	.81
Paraplegia	1.43 (1.17–1.75)	<.001	1.22 (0.99–1.49)	.06
Stroke	1.22 (1.06–1.40)	.006	1.10 (0.96–1.26)	.15

*Note*: Model c‐statistic for fixed effects‐only model = 0.70467.

Repair within 14 days continued to be more likely in the presence of extremity ischemia (aRR 1.26, 95% CI 1.05–1.50), rupture (aRR 1.32, 95% CI 1.14–1.51), shock (aRR 1.45, 95% CI 1.26–1.68), and acidosis (aRR 1.29, 95% CI 1.11–1.49). After adjustment, paraplegia and stroke were no longer associated with repair.

## 4 DISCUSSION

We aimed to describe patients presenting with TBAD, compare patients undergoing open repair, TEVAR, and no repair within 14 days of admission, and analyze factors associated with the presence or absence of repair within 14 days of admission. We found that most TBAD patients did not undergo repair within 14 days and that the relative proportion of repair within 14 days of admission decreased over time. Patients undergoing repair were younger, more likely to be male, and less likely to have comorbidities. The relative proportion of repair within 14 days and repair type varied regionally. Patients who underwent TEVAR more commonly had codes for rupture and extremity ischemia; patients who underwent open repair more commonly had codes for stroke, acidosis, and shock. After adjustment, male sex, weekday admission, peripheral vascular disease, extremity ischemia, rupture, shock, and acidosis were associated with increased likelihood of repair; older age, Middle Atlantic region, diabetes mellitus, antiplatelet use, and more recent year were associated with decreased likelihood of repair.

Among this cohort of 2613 TBAD patients including those with and without rupture and malperfusion, 38% of patients underwent repair within 14 days and 62% did not. This is similar to repair frequencies reported in other mixed “complicated” and “uncomplicated” TBAD cohorts, including Carroll et al (37% repaired) and Pape (37% repaired) but higher than in other reports, including Tsai (22% repaired) and Wang (13.1% repaired).^3^, ^5^, ^8^, ^14^ However, in our cohort, the frequency of repair versus medical management decreased over time (from 43% in 2014 to 26.4% in 2017), which conflicts with earlier reports by Pape et al using the International Registry of Acute Aortic Dissection (IRAD, 24.6% in 1995–1999 to 43.5% in 2010–2013), Carroll et al using the National Readmissions Database (31.9% in 2010 to 39.5% in 2014), and Wang et al using the Nationwide Inpatient Sample (16.2% in 2000 to 30.6% in 2012).

It is not clear why the overall frequency of repair decreased over time in the cohort. While the MarketScan dataset size also decreased over time, the incidence of TBAD remained relatively constant at about 15–20 per 100 000 enrolled records, similar to the incidence reported by Mody et al in CMS data.^15^ Our analysis uses the most recent nationally‐representative cohort that has been published (all others having used data prior to 2014). The association between year of index admission and lower likelihood of repair persisted on multivariable analysis, suggesting that it was not due to changes in patient characteristics over the study period. It may be that unmeasured factors, such as the change in clinician training paradigms, procedural reimbursement, or centralization of aortic care, are contributing to the observed trend. For instance, we were specifically interested in repair within 14 days; it is possible that repairs occurred with the same frequency each year, but at later times following the index presentation. A preference for subacute repair may also have increased over the study period following the 2014 publication of data from the VIRTUE Registry that showed that patients undergoing subacute TEVAR experienced lower mortality than the acute group while retaining the favorable aortic remodeling characteristics associated with acute repair.^16^ It is also possible that increased focus on the role of antihypertensive and anti‐impulse therapies in medical management contributed to decreased operative management of TBAD over time as. Clearly, other analyses in independent datasets are needed in order to contextualize this finding.

In our analysis, 66% of repairs within 14 days were open and 34% were TEVAR overall. However, the relative proportion of TEVAR versus open repair changed dramatically over the study period from 28.4% of early repairs in 2014 to 52.7% of early repairs in 2017. Our findings fall within range of previous studies, which have reported variable but increasing relative proportions of TEVAR versus open repair over time, including Wang (0.5% in 2000 to 44.9% in 2012), Sachs et al (6.6% of repairs in 2005 to 32.7% of repairs in 2007), and Carroll et al (44.1% in 2010 to 46.0% in 2014).^3^, ^4^, ^5^, ^6^, ^7^, ^8^ This reflects the generally increasing preference for endovascular approaches and advancement in endovascular techniques over time. The relatively high percentage of open repairs in our analysis may also reflect the fact that, despite excluding patients with any dissection admission in the year prior to the index, the study cohort likely includes chronic TBAD patients as well. This same feature of our study cohort may also provide some rationale for the decrease in open repairs observed over the study period: patients with chronic dissections may be more likely to require open repairs, and it is more likely that some patients with chronic dissections could have been included earlier in the study period, given our 1 year look‐back period.

Hypertension was the most common comorbidity in this cohort (present in 67.7% of patients overall 90 days prior to index), in keeping with its high rate nationally and its known association with aortic dissection. Other authors have reported slightly higher proportions of hypertension in TBAD cohorts ranging from 75.1% to 88.1%.^3^, ^5^, ^8^, ^14^, ^17^ It is possible that relatively fewer patients in our cohort had aortic dissection due to hypertension and relatively more had dissection due to trauma or connective tissue disorders because we did not specifically exclude patients with trauma or connective tissue disorders and included patients as young as 18 (though only 6.1% of patients were aged ≤40). Peripheral vascular disease was the second most common comorbidity (present in 46.4% of patients overall). This frequency is higher than has been reported in other TBAD cohorts, though the coding definition of peripheral vascular disease we used included cerebrovascular, aortic, visceral, and lower‐extremity atherosclerosis, which may explain this difference. It is also important to note that both hypertension and especially peripheral vascular disease were significantly under‐treated in this cohort. Among patients with diagnosed hypertension, 25.17% were not taking antihypertensive medications, and 60.56% of peripheral vascular disease patients were not taking statin medications. Only 4.9% of the overall cohort was taking antiplatelet medications, another important component of guideline‐based therapy for patients with atherosclerotic disease, though it should be noted that this does not account for patients taking aspirin over the counter. Future investigation including similar patients who did not develop TBAD may help illuminate the extent to which adequate treatment of hypertension and peripheral vascular disease contribute to TBAD incidence. Of note, there were 91 patients with rupture in our cohort who did not undergo any early repair. It is possible that these patients died prior to undergoing repair: unfortunately, mortality data are not available in MarketScan, though we required 14 days of enrollment following index date to try to exclude patients who had died. Finally, 46.2% of patients undergoing repair within 14 days of index admission did not have any codes for complications, suggesting that a significant number of the repairs were in patients with uncomplicated TBAD.

There are several limitations of this analysis. First, we were unable to describe and account for hospital‐ and clinician‐related data, including the TBAD treatment patterns of institutions, because the necessary data fields either had very high rates of missingness or very homogenous data (such as the provider type very frequently being listed as “acute care hospital” rather than a clinician specialty). We believe that institution‐level practice patterns likely significantly influence whether or not patients undergo early repair, but are unable to show that based off our data. Second, as noted above, the use of administrative data makes distinguishing between complications of TBAD versus complications of repair impossible, therefore we were not able to analyze repair patterns separately among patients with complicated and uncomplicated TBAD. Third, MarketScan includes only patients with certain insurance types from participating insurance carriers but prior research has shown TBAD to disproportionately affect underinsured patients, suggesting that there is an important group of TBAD patients who were not captured in this analysis.^18^ Fourth, MarketScan does not include mortality data. Although we required 14 days of continuous enrollment post‐index to ascertain early repair, we cannot be certain absolutely certain whether patients without early repair died before repair could be done, and we were not able to determine mortality following discharge, which prevented us from reporting post‐discharge complications. Fifth, because we were limited in the amount of data we had available for a look‐back period, this cohort represents a mix of patients with acute, sub‐acute, and chronic TBAD. While it therefore cannot illuminate practice patterns specifically among patients with acute TBAD, which is an area of active investigation nationally and internationally, we feel that an updated look at TBAD management patterns overall provides important context to ongoing scholarly activities and debate.

## 5 CONCLUSIONS

One third of patients presenting with TBAD in the MarketScan dataset between 2014 and 2017 underwent repair within 14 days of admission. The frequency of repair decreased over the study period due to decreasing open repairs; TEVAR became relatively more common compared to open repair. Older patients and those in the Middle Atlantic region were less likely to undergo repair, while male patients and those with certain complications were more likely to undergo repair. Further investigation into provider‐ and hospital‐specific factors as they relate to likelihood of repair is needed.

## CONFLICT OF INTEREST

Sreekanth Vemulapalli: Research support: American College of Cardiology, Society of Thoracic Surgeons, Food and Drug Administration (NEST), National Institutes of Health, Boston Scientific. Consulting/advisory board: Heartflow, Janssen, Boston Scientific, American College of Physicians. R. Patel: Research grants: Janssen, Bayer, NHLBI, Heartflow, Phillips, advisory board/consulting: Bayer, Janssen, Heartflow. W. Schuyler Jones: Research support – Boehringer Ingelheim, Doris Duke Charitable Foundation, National Institutes of Health, Patient‐Centered Outcomes Research Institute; advisory board – Bayer, Bristol‐Myers Squibb, Janssen Pharmaceuticals. The content is solely the responsibility of the authors and does not necessarily represent the official views of the National Institutes of Health.

## Supporting information

**Appendix S1**: Supporting information.

## Data Availability

The data that support the findings of this study are part of the MarketScan database, a proprietary dataset, and are therefore not able to be shared.
