# Supplementary material for: National trends in repair for type B aortic dissection
Source: Clin Cardiol. 2021 Jun 26;44(8):1058–68. doi: 10.1002/clc.23672 (PMC8364733; doi:10.1002/clc.23672)
Supplement: Supplementary file 1 — Appendix S1: Supporting information. [file CLC-44-1058-s001.zip › CLC_23672_CLC_23672_Appendix A.docx]

**Appendix A. Exclusionary diagnosis and procedure codes**

| **Diagnosis** | | **ICD-9-CM/PCS** | **ICD-10-CM/PCS** | **CPT** |
| --- | --- | --- | --- | --- |
| Procedures indicating Type A ascending dissection | Cardioplegia and cardiopulmonary bypass | 39.61, 39.63, 39.66 | 3E080GC | 33946, 33947, 33948, 33949, 33951, 33952, 33953, 33954, 33955, 33956, 33957, 33958, 33959, 33960, 33961, 33962, 33963, 33964, 33965, 33966, 33969, 33984, 33985, 33986, 33987, 33988, 33989, 33990, 33991, 33992, 33993, 36822, 92970, 92971, 0048T, 0049T, 0050T |
|  | Valve repair or replacement | 35.00 – 35.99 | 0240**, 027F0**, 02QF0Z*, 02ZF4Z**, 02UF4**, 02BX0ZZ, 02BX3ZZ, 02BX4ZZ | 33361-33369, 33400-33406, 33410-33420, 33422, 33425-33427, 33430, 33440, 33460, 33463-33465, 33468, 33470-33472, 33474-33478, 33496, 33600, 33602, 33684, 33732, 33860, 33863, 33864, 33866, 33920, 33391, 33390, 33330, 33335, 92986, 92987, 92990, 0256T, 0257T, 0258T, 0259T, 0262T, 0318T, 0343T, 0344T, 0345T, 33999 |
|  | Operations on vessels of the heart | 36.00 – 36.99 | 021009*, 2100A*, 02100J*, 02100K*, 02100Z*, 021049*, 02104A*, 02104J*, 02104K*, 02104Z*, 021109*, 02110A*, 02110J*, 02110K*, 02110Z*, 021149*, 02114A*, 02114J*, 02114K*, 02114Z*, 021209*, 02120A*, 02120J*, 02120K*, 02120Z*, 021309*, 02130A*, 02130J*, 02130K*, 02130Z*, 021349*, 02134A*, 02134J*, 02134K*, 02134Z* | 33572, 33510-33514, 33516-33519, 33521-33523, 33530, 33533, 33534, 33535, 33536, 35600, S2205, S2206, S2207, S2208, S2209 |
|  | Other procedures (e.g., ascending thoracic aortic procedures, donor heart procurement)/discontinued codes | 37.0 | 02BX0ZZ, 02BX3ZZ, 02BX4ZZ | 32160, 32658-32661, 33015, 33020, 33025, 33030, 33031, 33050, 33120, 33130, 33250, 33251, 33253, 33254, 33255, 33256, 33257, 33258, 33259, 33261, 33265, 33266, 33300, 33305, 33310, 33315, 33500-33507, 33542, 33545, 33548, 33608, 33610, 33611, 33612, 33615, 33617, 33619, 33620, 33621, 33622, 33641, 33645, 33647, 33660, 33665, 33670, 33675, 33676, 33677, 33681, 33688, 33692, 33694, 33697, 33702, 33710, 33720, 33722, 33724, 33726, 33730, 33735-33737, 33770, 33771, 33774-33783, 33786, 33813, 33814, 33924, 33940, 0024T, 0051T, 0052T, 0053T, 0166T, 0167T, 0302T, 0303T, 0304T, 0305T, 0306T, 0307T, 92975, 92977, 33010, 33011, 33140, 33141, 92992, 92993, 93580, 93581, 93582, 93583 |
