# Supplementary material for: National trends in repair for type B aortic dissection
Source: Clin Cardiol. 2021 Jun 26;44(8):1058–68. doi: 10.1002/clc.23672 (PMC8364733; doi:10.1002/clc.23672)
Supplement: Supplementary file 1 — Appendix S1: Supporting information. [file CLC-44-1058-s001.zip › CLC_23672_CLC_23672_Appendix B.docx]

**Appendix B: Generic names of medications for defining medication therapy at index presentation**

| **THRCLDS** | **GENNME** | **GENERID** |
| --- | --- | --- |
| Analgesics/Antipyretics, NEC | Clonidine Hydrochloride | 128443 |
| Analgesics/Antipyretics, NEC | Clonidine Hydrochloride;Clonidine Hydrochloride | 130123 |
| Antidiabetic Agents, Insulins | Insulin Aspart Protamine/Insulin Aspart | 119317 |
| Antidiabetic Agents, Insulins | Insulin Aspart, Recombinant | 118375, 134369, 134371 |
| Antidiabetic Agents, Insulins | Insulin Beef Isophane (NPH) | 117034 |
| Antidiabetic Agents, Insulins | Insulin Beef Regular/Insulin Pork Isophane (NPH) | 108269 |
| Antidiabetic Agents, Insulins | Insulin Beef Regular/Insulin Pork Regular | 108267 |
| Antidiabetic Agents, Insulins | Insulin Beef Regular/Insulin Pork Zinc (Lente) | 108268 |
| Antidiabetic Agents, Insulins | Insulin Beef Zinc (Lente) | 117035 |
| Antidiabetic Agents, Insulins | Insulin Beef Zinc, Extended (Ultralente) | 108266 |
| Antidiabetic Agents, Insulins | Insulin Beef Zinc, Prompt (Semilente) | 117036 |
| Antidiabetic Agents, Insulins | Insulin Degludec | 130470, 132067 |
| Antidiabetic Agents, Insulins | Insulin Degludec/Liraglutide | 131548 |
| Antidiabetic Agents, Insulins | Insulin Detemir | 124922 |
| Antidiabetic Agents, Insulins | Insulin Glargine, Recombinant | 117339, 131672 |
| Antidiabetic Agents, Insulins | Insulin Glargine, Recombinant/Lixisenatide | 133491 |
| Antidiabetic Agents, Insulins | Insulin Glulisine | 126770 |
| Antidiabetic Agents, Insulins | Insulin Human Inhaled | 124985, 124989, 131253, 131254, 131874 |
| Antidiabetic Agents, Insulins | Insulin Human Inhaled;Insulin Human Inhaled | 125241, 131570, 131875, 133581 |
| Antidiabetic Agents, Insulins | Insulin Human Isophane (NPH) | 106630, 999999 |
| Antidiabetic Agents, Insulins | Insulin Human Isophane (NPH)/Insulin Human Regular | 106633, 106634, 999999 |
| Antidiabetic Agents, Insulins | Insulin Human Regular | 106632, 111530, 132362, 999999 |
| Antidiabetic Agents, Insulins | Insulin Human Regular, Buffered | 123541 |
| Antidiabetic Agents, Insulins | Insulin Human Zinc (Lente) | 106631, 999999 |
| Antidiabetic Agents, Insulins | Insulin Human Zinc, Extended (Ultralente) | 107660 |
| Antidiabetic Agents, Insulins | Insulin Lispro Protamine/Insulin Lispro | 114252, 124987 |
| Antidiabetic Agents, Insulins | Insulin Lispro, Recombinant | 110249, 131890, 134502 |
| Antidiabetic Agents, Insulins | Insulin Pork Isophane (NPH) | 107902 |
| Antidiabetic Agents, Insulins | Insulin Pork Regular | 107903, 108237 |
| Antidiabetic Agents, Insulins | Insulin Pork Zinc (Lente) | 107901 |
| Antidiabetic Agents, Insulins | Insulin, Beef | 999999 |
| Antidiabetic Agents, Insulins | Insulin, Beef/Pork | 999999 |
| Antidiabetic Agents, Insulins | Insulin, Human Regular Buffered | 108708 |
| Antidiabetic Agents, Insulins | Insulin, Pork | 999999 |
| Antiplatelet Agents, NEC | Aspirin;Clopidogrel Hydrogen Sulfate | 134283 |
| Antiplatelet Agents, NEC | Clopidogrel Hydrogen Sulfate | 112655, 126480 |
| Antiplatelet Agents, NEC | Prasugrel Hydrochloride | 127598, 127599 |
| Antiplatelet Agents, NEC | Ticagrelor | 129110, 132039 |
| Antiplatelet Agents, NEC | Vorapaxar | 131163 |
| Cardiac Drugs, NEC | Aliskiren/Amlodipine Besylate | 128347, 128348, 128349, 128350 |
| Cardiac Drugs, NEC | Aliskiren/Amlodipine Besylate/Hydrochlorothiazide | 128647, 128648, 128649, 128650, 128651 |
| Cardiac Drugs, NEC | Aliskiren/Hydrochlorothiazide | 126432, 126435, 126441, 126442 |
| Cardiac Drugs, NEC | Aliskiren/Valsartan | 127759, 127762 |
| Cardiac Drugs, NEC | Amlodipine Besylate/Atorvastatin Calcium | 123733, 123799, 123801, 123803, 123804, 123805, 123807, 123808, 124543, 124545, 124546 |
| Cardiac Drugs, NEC | Amlodipine Besylate/HCTZ/Olmesartan Medoxomil | 128299, 128300, 128302, 128303, 128304 |
| Cardiac Drugs, NEC | Azilsartan Medoxomil | 128770, 128772 |
| Cardiac Drugs, NEC | Azilsartan Medoxomil/Chlorthalidone | 129470, 129471 |
| Cardiac Drugs, NEC | Candesartan Cilexetil | 113366, 113367, 113368, 113369 |
| Cardiac Drugs, NEC | Candesartan Cilexetil/Hydrochlorothiazide | 114928, 114929, 127164 |
| Cardiac Drugs, NEC | Eprosartan Mesylate | 114047, 114048 |
| Cardiac Drugs, NEC | Eprosartan Mesylate/Hydrochlorothiazide | 122846, 122848 |
| Cardiac Drugs, NEC | Hydrochlorothiazide/Irbesartan | 113845, 113846, 124701 |
| Cardiac Drugs, NEC | Hydrochlorothiazide/Losartan Potassium | 108280, 113490, 124848 |
| Cardiac Drugs, NEC | Hydrochlorothiazide/Olmesartan Medoxomil | 123287, 123288, 123290 |
| Cardiac Drugs, NEC | Hydrochlorothiazide/Telmisartan | 116804, 116805, 124124 |
| Cardiac Drugs, NEC | Hydrochlorothiazide/Valsartan | 112950, 112951, 119178, 125131, 125133 |
| Cardiac Drugs, NEC | Irbesartan | 112221, 112222, 112223 |
| Cardiac Drugs, NEC | Losartan Potassium | 108264, 108265, 113580 |
| Cardiac Drugs, NEC | Nebivolol/Valsartan | 132815 |
| Cardiac Drugs, NEC | Olmesartan Medoxomil | 119186, 119187, 119188 |
| Cardiac Drugs, NEC | Sacubitril/Valsartan | 131870, 131871, 131872 |
| Cardiac Drugs, NEC | Telmisartan | 113494, 113495, 115414 |
| Cardiac Drugs, NEC | Valsartan | 111339, 111577, 118239, 118613, 118614, 119273 |
| Cardiac, ACE Inhibitors | Amlodipine/Perindopril Arginine | 131621, 131622, 131623 |
| Cardiac, ACE Inhibitors | Benazepril Hydrochloride | 100303, 100304, 100305, 100306 |
| Cardiac, ACE Inhibitors | Benazepril Hydrochloride/Hydrochlorothiazide | 104193, 104194, 104195, 104196 |
| Cardiac, ACE Inhibitors | Captopril | 100500, 100501, 100502, 100503 |
| Cardiac, ACE Inhibitors | Captopril/Hydrochlorothiazide | 100504, 100505, 100506, 100507 |
| Cardiac, ACE Inhibitors | Enalapril Maleate | 101332, 101333, 101334, 101335, 130755, 133221 |
| Cardiac, ACE Inhibitors | Enalapril Maleate/Felodipine | 111807, 113579 |
| Cardiac, ACE Inhibitors | Enalapril Maleate/Hydrochlorothiazide | 101337, 108736 |
| Cardiac, ACE Inhibitors | Fosinopril Sodium | 101638, 101639, 108822 |
| Cardiac, ACE Inhibitors | Fosinopril Sodium/Hydrochlorothiazide | 114576, 114577 |
| Cardiac, ACE Inhibitors | Hydrochlorothiazide/Lisinopril | 102236, 102237, 102238 |
| Cardiac, ACE Inhibitors | Hydrochlorothiazide/Moexipril Hydrochloride | 112166, 112169, 119117 |
| Cardiac, ACE Inhibitors | Hydrochlorothiazide/Quinapril Hydrochloride | 114573, 114574, 114575 |
| Cardiac, ACE Inhibitors | Lisinopril | 102231, 102232, 102233, 102234, 102235, 113749, 133042 |
| Cardiac, ACE Inhibitors | Lisinopril;Medical Food | 128935, 129020, 131166 |
| Cardiac, ACE Inhibitors | Moexipril Hydrochloride | 108322, 108323 |
| Cardiac, ACE Inhibitors | Perindopril Erbumine | 114013, 114014, 114015 |
| Cardiac, ACE Inhibitors | Quinapril Hydrochloride | 103367, 103368, 103369, 103370, 999999 |
| Cardiac, ACE Inhibitors | Ramipril | 103387, 103388, 103389, 103390, 126304, 126305, 126306, 126309 |
| Cardiac, ACE Inhibitors | Trandolapril | 109825, 109826, 109827 |
| Cardiac, ACE Inhibitors | Trandolapril/Verapamil Hydrochloride | 111335, 111336, 111337, 111338 |
| Cardiac, Alpha-Beta Blockers | Hydrochlorothiazide/Labetalol Hydrochloride | 109519, 109520, 109632, 999999 |
| Cardiac, Beta Blockers | Acebutolol Hydrochloride | 100002, 100003, 999999 |
| Cardiac, Beta Blockers | Atenolol | 100240, 100241, 100242, 999999 |
| Cardiac, Beta Blockers | Atenolol/Chlorthalidone | 100243, 100244 |
| Cardiac, Beta Blockers | Bendroflumethiazide/Nadolol | 102628, 102629 |
| Cardiac, Beta Blockers | Bisoprolol Fumarate | 104117, 104118 |
| Cardiac, Beta Blockers | Bisoprolol Fumarate/Hydrochlorothiazide | 104119, 104120, 104121 |
| Cardiac, Beta Blockers | Carvedilol | 111954, 111955, 111956, 111957 |
| Cardiac, Beta Blockers | Carvedilol Phosphate | 125476, 125478, 125479, 125480 |
| Cardiac, Beta Blockers | Carvedilol;Medical Food | 128944 |
| Cardiac, Beta Blockers | Hydrochlorothiazide/Metoprolol Succinate | 125286, 125287, 125290 |
| Cardiac, Beta Blockers | Hydrochlorothiazide/Metoprolol Tartrate | 102502, 102503, 102504 |
| Cardiac, Beta Blockers | Hydrochlorothiazide/Propranolol Hydrochloride | 103306, 103307, 103308, 103309, 103310, 999999 |
| Cardiac, Beta Blockers | Hydrochlorothiazide/Timolol Maleate | 103823 |
| Cardiac, Beta Blockers | Penbutolol Sulfate | 102902 |
| Cardiac, Beta Blockers | Pindolol | 103063, 103064 |
| Cardiac, Beta Blockers | Propranolol Hydrochloride | 103293, 103294, 103295,103296, 103297, 103298, 103299, 103300, 103301, 103302, 103303, 103305, 109597, 131084, 999999 |
| Cardiac, Beta Blockers | Propranolol/Scopolamine | 131500 |
| Cardiac, Beta Blockers | Timolol Maleate | 103826, 103827, 103828, 999999 |
| Cardiac, Calcium Channel | Amlodipine Besylate | 100156, 100157, 100158 |
| Cardiac, Calcium Channel | Amlodipine Besylate/Benazepril Hydrochloride | 108398, 108399, 108400, 119223, 125145, 125146 |
| Cardiac, Calcium Channel | Amlodipine Besylate/Hydrochlorothiazide/Valsartan | 127384, 127386, 127388, 127389, 127397 |
| Cardiac, Calcium Channel | Amlodipine Besylate/Olmesartan Medoxomil | 126171, 126173, 126174, 126175 |
| Cardiac, Calcium Channel | Amlodipine Besylate/Telmisartan | 127827, 127828, 127829, 127830 |
| Cardiac, Calcium Channel | Amlodipine Besylate/Valsartan | 125997, 125998, 126000, 126001 |
| Cardiac, Calcium Channel | Amlodipine Besylate;Medical Food | 128949 |
| Cardiac, Calcium Channel | Diltiazem Hydrochloride | 101208, 101209, 101210, 101211, 101212, 101213, , 101216, 101218, 109080, 113500, 114166, 114168, 114169, 114170, 114171, 114172, 114173, 114174, 114175, 122983, 122984, 122985, 122987, 122988, 122989, 134877, 134878, 134879, 134880, 134881 |
| Cardiac, Calcium Channel | Diltiazem Malate | 112947, 112948, 112949 |
| Cardiac, Calcium Channel | Diltiazem Malate/Enalapril Maleate | 112316 |
| Cardiac, Calcium Channel | Felodipine | 101513, 101514, 101515 |
| Cardiac, Calcium Channel | Isradipine | 102113, 102114, 111362, 111363 |
| Cardiac, Calcium Channel | Nicardipine Hydrochloride | 102704, 102706, 102707, 102708, 102709 |
| Cardiac, Calcium Channel | Nifedipine | 102718, 102719, 102720, 102721, 102722, 999999 |
| Cardiac, Calcium Channel | Nisoldipine | 109217, 109219, 109220, 109222, 126538, 126539, 126541, 126543 |
| Cardiac, Calcium Channel | Verapamil Hydrochloride | 104004, 104005, 104006, 104007, 104008, 104009, 104010, , 104012, 104013, 109907, 113803, 113804, 113805, 114176, 114177, |
| Coag/Anticoag, Anticoagulants | Apixaban | 130300, 130318 |
| Coag/Anticoag, Anticoagulants | Dabigatran Etexilate Mesylate | 128520, 128521, 132332 |
| Coag/Anticoag, Anticoagulants | Edoxaban | 131602, 131603, 131604 |
| Coag/Anticoag, Anticoagulants | Rivaroxaban | 128994, 129283, 129284, 134932 |
| Coag/Anticoag, Anticoagulants | Rivaroxaban;Rivaroxaban | 131456 |
| Coag/Anticoag, Anticoagulants | Warfarin Sodium | 104045, 104046, 104047, 104048, 104049, 104050, 104051, , 109779, 111798, 999999 |
| Diuretics, Loop Diuretics | Benzalkonium Chloride;Furosemide | 130959 |
| Diuretics, Loop Diuretics | Bumetanide | 100415, 100416, 100417, 999999 |
| Diuretics, Loop Diuretics | Furosemide | 101646, 101647, 101649, 101650, 101651, 101652 |
| Diuretics, Loop Diuretics | Furosemide/Sodium Chloride | 133327 |
| Diuretics, Loop Diuretics | Torsemide | 104167, 104168, 104169, 104170, 104171 |
| Diuretics, Potassium-Sparing | Amiloride Hydrochloride/Hydrochlorothiazide | 100089, 999999 |
| Diuretics, Potassium-Sparing | Hydrochlorothiazide/Spironolactone | 103576, 103577, 999999 |
| Diuretics, Potassium-Sparing | Hydrochlorothiazide/Triamterene | 103894, 103895, 103896, 103897, 103898, 999999 |
| Diuretics, Potassium-Sparing | Spironolactone | 103573, 103574, 103575, 134178, 999999 |
| Diuretics, Potassium-Sparing | Triamterene | 103892, 103893, 999999 |
| Diuretics, Thiazides & Related | Bendroflumethiazide | 100307, 100308, 999999 |
| Diuretics, Thiazides & Related | Chlorothiazide | 100697, 100698, 100699, 999999 |
| Diuretics, Thiazides & Related | Chlorothiazide Sodium | 108148 |
| Diuretics, Thiazides & Related | Chlorthalidone | 100802, 100803, 100804, 100805, 999999 |
| Diuretics, Thiazides & Related | Cryptenamine/Methyclothiazide | 999999 |
| Diuretics, Thiazides & Related | Hydrochlorothiazide | 101814, 101815, 101816, 101817, 111511, 125835 |
| Diuretics, Thiazides & Related | Indapamide | 101991, 101992, 999999 |
| Diuretics, Thiazides & Related | Kcl/Bendroflumethiazide | 999999 |
| Diuretics, Thiazides & Related | Methyclothiazide | 102444, 102445, 999999 |
| Diuretics, Thiazides & Related | Metolazone | 102498, 102499, 102500, 102501, 999999 |
| Hypotensive Agents, NEC | Chlorothiazide/Methyldopa | 102456, 102457, 999999 |
| Hypotensive Agents, NEC | Chlorothiazide/Reserpine | 100700, 100701, 999999 |
| Hypotensive Agents, NEC | Chlorthalidone/Clonidine Hydrochloride | 100889, 100890, 100891, 999999 |
| Hypotensive Agents, NEC | Chlorthalidone/Reserpine | 100806, 100807, 999999 |
| Hypotensive Agents, NEC | Clonidine | 100886, 100887, 100888, 128642, 128643 |
| Hypotensive Agents, NEC | Clonidine Hydrochloride | 100892, 100893, 100894, 999999 |
| Hypotensive Agents, NEC | Deserpidine/Hydrochlorothiazide | 101014, 101015, 101016 |
| Hypotensive Agents, NEC | Deserpidine/Methyclothiazide | 102446, 102447, 999999 |
| Hypotensive Agents, NEC | Diazoxide | 101143, 101144, 101145, 999999 |
| Hypotensive Agents, NEC | Guanethidine Monosulfate/Hydrochlorothiazide | 101724, 999999 |
| Hypotensive Agents, NEC | Guanfacine Hydrochloride | 101727, 101728, 127736, 127737, 127738, 127739 |
| Hypotensive Agents, NEC | Hydralazine Hydrochloride | 101804, 101805, 101806, 101807, 101808, 999999 |
| Hypotensive Agents, NEC | Hydralazine Hydrochloride/Hydrochlorothiazide | 101809, 101810, 101811, 109525, 999999 |
| Hypotensive Agents, NEC | Hydralazine Hydrochloride/Reserpine | 999999 |
| Hypotensive Agents, NEC | Hydrochlorothiazide/Methyldopa | 102458, 102459, 102460, 102461, 999999 |
| Hypotensive Agents, NEC | Hydrochlorothiazide/Reserpine | 101818, 101819, 999999 |
| Hypotensive Agents, NEC | Methyclothiazide/Reserpine | 102448 |
| Hypotensive Agents, NEC | Methyldopa | 102452, 102453, 102454, 102455, 999999 |
| Hypotensive Agents, NEC | Methyldopate Hydrochloride | 102462, 999999 |
| Hypotensive Agents, NEC | Minoxidil | 102548, 102550, 999999 |
| Hypotensive Agents, NEC | Phenoxybenzamine Hydrochloride | 102982 |
| Hypotensive Agents, NEC | Phentolamine Mesylate | 108092, 125149, 126731, 999999 |
| Hypotensive Agents, NEC | Polythiazide/Prazosin Hydrochloride | 103193, 103194, 103195 |
| Hypotensive Agents, NEC | Prazosin Hydrochloride | 103190, 103191, 103192, 999999 |
| Hypotensive Agents, NEC | Terazosin Hydrochloride | 103639, 103640, 103641, 103642, 108048, 108049, 108050, 108051, 116788, 116789, 116790, 116791 |
| Misc Therapeutic Agents, NEC | Alfuzosin Hydrochloride | 123450 |
| Misc Therapeutic Agents, NEC | Dutasteride/Tamsulosin Hydrochloride | 128242 |
| Misc Therapeutic Agents, NEC | Silodosin | 127022, 127023 |
| Misc Therapeutic Agents, NEC | Tamsulosin Hydrochloride | 112106, 111542, 114352, 124635, 124636, 124637, 128183 |
| Muscle Relax, Skeletal Central | Tizanidine Hydrochloride | 111542, 114352, 124635, 124636, 124637 |
| Muscle Relax, Skeletal Central | Tizanidine Hydrochloride;Gel, Multi Ingredient | 128183 |
| Pharmaceutical Aids/Adjuv, NEC | Acebutolol Hydrochloride | 108927 |
| Pharmaceutical Aids/Adjuv, NEC | Amlodipine Besylate | 127123 |
| Pharmaceutical Aids/Adjuv, NEC | Atenolol | 108897 |
| Pharmaceutical Aids/Adjuv, NEC | Benazepril Hydrochloride | 130550 |
| Pharmaceutical Aids/Adjuv, NEC | Bendroflumethiazide | 108894, 109254 |
| Pharmaceutical Aids/Adjuv, NEC | Bumetanide | 108443 |
| Pharmaceutical Aids/Adjuv, NEC | Captopril | 118643 |
| Pharmaceutical Aids/Adjuv, NEC | Clonidine Hydrochloride | 104788 |
| Pharmaceutical Aids/Adjuv, NEC | Diazoxide | 108911 |
| Pharmaceutical Aids/Adjuv, NEC | Diltiazem Hydrochloride | 108923 |
| Pharmaceutical Aids/Adjuv, NEC | Enalapril Maleate | 123170 |
| Pharmaceutical Aids/Adjuv, NEC | Furosemide | 108848, 109024 |
| Pharmaceutical Aids/Adjuv, NEC | Guanfacine Hydrochloride | 124349 |
| Pharmaceutical Aids/Adjuv, NEC | Hydralazine Hydrochloride | 104820 |
| Pharmaceutical Aids/Adjuv, NEC | Hydrochlorothiazide | 104944 |
| Pharmaceutical Aids/Adjuv, NEC | Indapamide | 108866 |
| Pharmaceutical Aids/Adjuv, NEC | Insulin Bovine | 123240, 124210 |
| Pharmaceutical Aids/Adjuv, NEC | Lisinopril | 124046 |
| Pharmaceutical Aids/Adjuv, NEC | Methyclothiazide | 108870 |
| Pharmaceutical Aids/Adjuv, NEC | Methyldopa | 108869, 109051 |
| Pharmaceutical Aids/Adjuv, NEC | Minoxidil | 113506 |
| Pharmaceutical Aids/Adjuv, NEC | Nicardipine Hydrochloride | 118799 |
| Pharmaceutical Aids/Adjuv, NEC | Nifedipine | 105539 |
| Pharmaceutical Aids/Adjuv, NEC | Olmesartan Medoxomil | 134150 |
| Pharmaceutical Aids/Adjuv, NEC | Phenoxybenzamine Hydrochloride | 113507 |
| Pharmaceutical Aids/Adjuv, NEC | Phentolamine Mesylate | 111429 |
| Pharmaceutical Aids/Adjuv, NEC | Pindolol | 108891 |
| Pharmaceutical Aids/Adjuv, NEC | Prazosin Hydrochloride | 108930, 109064 |
| Pharmaceutical Aids/Adjuv, NEC | Propranolol Hydrochloride | 104524 |
| Pharmaceutical Aids/Adjuv, NEC | Spironolactone | 104692 |
| Pharmaceutical Aids/Adjuv, NEC | Spironolactone, Micronized | 123387 |
| Pharmaceutical Aids/Adjuv, NEC | Terazosin Hydrochloride | 123253 |
| Pharmaceutical Aids/Adjuv, NEC | Timolol Maleate | 108628 |
| Pharmaceutical Aids/Adjuv, NEC | Tizanidine Hydrochloride | 130777 |
| Pharmaceutical Aids/Adjuv, NEC | Tolazoline Hydrochloride | 124195, 127467 |
| Pharmaceutical Aids/Adjuv, NEC | Triamterene | 108844 |
| Pharmaceutical Aids/Adjuv, NEC | Verapamil Hydrochloride | 108857 |
| Pharmaceutical Aids/Adjuv, NEC | Warfarin Sodium | 108856, 123258 |
| Vasodilating Agents, NEC | Hydralazine Hydrochloride/Isosorbide Dinitrate | 124752 |
| Vasodilating Agents, NEC | Isosorbide Dinitrate | 102093, 102094, 102095, 102096, 102097, 102098, 102099, 102100, 102101, 102102, 102103, 108521, 108522, 999999 |
| Vasodilating Agents, NEC | Isosorbide Mononitrate | 102104, 102105, 102106, 108335, 109081 |
| Vasodilating Agents, NEC | Tolazoline Hydrochloride | 103848, 999999 |
|  | Acebutolol Hydrochloride | 108927 |
|  | Atenolol | 108897 |
|  | Bendroflumethiazide | 108894 |
|  | Bumetanide | 108443 |
|  | Clonidine Hydrochloride | 104788 |
|  | Diazoxide | 108911 |
|  | Diltiazem Hydrochloride | 101209, 101212, 101216, 101218, 108923 |
|  | Furosemide | 108848 |
|  | Hydralazine Hydrochloride | 104820 |
|  | Hydrochlorothiazide | 104944 |
|  | Indapamide | 108866 |
|  | Methyclothiazide | 108870 |
|  | Minoxidil | 113506 |
|  | Nifedipine | 105539 |
|  | Phentolamine Mesylate | 111429 |
|  | Pindolol | 103063, 103064, 108891 |
|  | Prazosin Hydrochloride | 108930, 104524 |
|  | Spironolactone | 104692 |
|  | Timolol Maleate | 108628 |
|  | Triamterene | 108844, 109076 |
|  | Verapamil Hydrochloride | 108857 |
|  | Warfarin Sodium | 108856 |
| Antihyperlipidemic Drugs, NEC | Alirocumab | 131904, 131905 |
| Antihyperlipidemic Drugs, NEC | Alirocumab |  |
| Antihyperlipidemic Drugs, NEC | Aspirin;Pravastatin Sodium | 123225, 123226, 123227, 123228, 123229, 123230 |
| Antihyperlipidemic Drugs, NEC | Atorvastatin Calcium | 111573, 111574, 111988, 114748, 131516 |
| Antihyperlipidemic Drugs, NEC | Atorvastatin Calcium/Ezetimibe | 130608, 130609, 130610, 130611 |
| Antihyperlipidemic Drugs, NEC | Cholestyramine | 100811, 100812, 109367, 109477, 110747, 114610, 999999, 119048 |
| Antihyperlipidemic Drugs, NEC | Colestipol Hydrochloride | 100930, 100931, 100932, 108090 |
| Antihyperlipidemic Drugs, NEC | Colestipol Hydrochloride, Micronized | 125989 |
| Antihyperlipidemic Drugs, NEC | Evolocumab | 131942, 133005 |
| Antihyperlipidemic Drugs, NEC | Ezetimibe | 122762 |
| Antihyperlipidemic Drugs, NEC | Ezetimibe/Simvastatin | 124153, 124154, 124155, 124157 |
| Antihyperlipidemic Drugs, NEC | Fenofibrate | 113106, 114029, 114564, 118178, 118397, 124474, 124476, 124700, 124966, 124967, 126506, 126508, 131120, 131121, 131517 |
| Antihyperlipidemic Drugs, NEC | Fenofibrate, Micronized | 119257, 119258, 119329, 124579, 124580, 130884, 130885 |
| Antihyperlipidemic Drugs, NEC | Fluvastatin Sodium | 104188, 104189, 115055 |
| Antihyperlipidemic Drugs, NEC | Gemfibrozil | 101656, 999999, 108847, 109026 |
| Antihyperlipidemic Drugs, NEC | Lomitapide Mesylate | 130304, 130305, 130308, 131803, 131804, 131806 |
| Antihyperlipidemic Drugs, NEC | Lovastatin | 102268, 102269, 102270, 119232, 119233, 123302, 123853 |
| Antihyperlipidemic Drugs, NEC | Lovastatin/Niacin | 119297, 119298, 119299, 125220 |
| Antihyperlipidemic Drugs, NEC | Niacin/Simvastatin | 126490, 126491, 126492, 128314, 128318 |
| Antihyperlipidemic Drugs, NEC | Pitavastatin | 134069, 134070, 134071 |
| Antihyperlipidemic Drugs, NEC | Pitavastatin Calcium | 127634, 127637, 127638 |
| Antihyperlipidemic Drugs, NEC | Pravastatin Sodium | 103186, 103187, 103188, 118907 |
| Antihyperlipidemic Drugs, NEC | Rosuvastatin Calcium | 123346, 123347, 123348, 123350 |
| Antihyperlipidemic Drugs, NEC | Simvastatin | 103469, 103470, 103471, 103472, 113213, 132974, 132975, 127509 |
| Antihyperlipidemic Drugs, NEC | Simvastatin/Sitagliptin Phosphate | 129225, 129226, 129227, 130211, 130212, 130213 |
| Cardiac Drugs, NEC | Amlodipine Besylate/Atorvastatin Calcium | 123733, 123799, 123801, 123803, 123804, 123805, 123807, 123808, 124543, 124545, 124546 |
| Pharmaceutical Aids/Adjuv, NEC | Cholestyramine Resin | 126360 |
