# Supplementary material for: National trends in repair for type B aortic dissection
Source: Clin Cardiol. 2021 Jun 26;44(8):1058–68. doi: 10.1002/clc.23672 (PMC8364733; doi:10.1002/clc.23672)
Supplement: Supplementary file 1 — Appendix S1: Supporting information. [file CLC-44-1058-s001.zip › CLC_23672_CLC_23672_Appendix C.docx]

**Appendix C. Code list for diagnoses indicating TBAD complications**

| **Diagnosis** | **ICD-9-CM** | **ICD-10-CM** |
| --- | --- | --- |
| Rupture | 441.1, 441.3, 441.5 | I71.1, I71.3, I71.5, I71.8 |
| Bowel ischemia | 557.0, 557.9 | K55.0, K55.01, K55.01, K55.012, K55.019, K55.021, K55.022, K55.029, K55.031, K55.032, K55.039, K55.041, K55.042, K55.049, K55.051, K55.052, K55.059, K55.06, K55.061, K55.062, K55.069 |
| Extremity ischemia | 444.21, 444.22, 444.81, 444.89, 444.9, 445.02 | I74.2, I74.3, I74.4, I74.5, I74.8, I74.9, I77.72 |
| Acidosis | 276.2, 276.4 | E87.2, E 87.4 |
| Shock | 276.50, 276.52, 785.5, 785.50, 785.51, 785.59 | E86.1, E86.9, R57.0, R57.1, R57.8, R57.9 |
| Renal artery dissection and/or renal failure | 443.23, 445.81, 584.5, 584.6, 584.7, 584.8, 584.9, 593.81 | N17.0, N17.1, N17.2, N17.8, N17.9, N28.0, I77.73 |
| Paraplegia | 336.1, 342.9, 342.90, 342.91, 342.92, 344.1, 344.30, 344.31, 344.32 344.89 | G95.11, G81.90, G81.91, G81.92, G82.20, G82.21, G82.22, G83.10, G83.11, G83.12, G83.13, G83.14, G83.82 |
| Stroke | 431, 432.0, 432.9, 433.01, 433.22, 433.21, 433.31, 433.81, 433.91, 434.01, 434.11, 434.91, 443.21, 443.24 | I61.0, I61.1, I61.2, I61.3, I61.4, I61.5, I61.6, I61.8, I61.9, I63.00, I63.01, I63.011, I63.012, I63.013, I63.019, I63.02, I63.03, I63.031, I63.032, I63.033, I63.039, I63.1, I63.10, I63.11, I63.111, I63.112, I63.113, I63.119, I63.12, I63.13, I63.131, I63.132, I63.133, I63.139, I63.19, I63.2, I63.20, I63.21, I63.211, I63.212, I63.213, I63.219, I63.22, I63.23, I63.231, I63.232, I63.233, I63.239, I63.29, I63.3, I63.30, I63.31, I63.311, I63.312, I63.313, I63.319, I63.32, I63.321, I63.322, I63.323, I63.329, I63.33, I63.331, I63.332, I63.333, I63.339, I63.34, I63.341, I63.342, I63.343, I63.349, I63.39, I63.4, I63.40, I63.41, I63.411, I63.412, I63.413, I63.419, I63.42, I63.421, I63.422, I63.423, I63.429, I63.43, I63.431, I63.432, I63.433, I63.439, I63.44, I63.441, I63.442, I63.443, I63.449, I63.49, I63.5, I63.50, I63.51, I63.511, I63.512, I63.513, I63.519, I63.52. I63.521, I63.522, I63.523, I63.529, I63.53, I63.531, I63.532, I63.533, I63.539, I63.54, I63.541, I63.542, I63.543, I63.549, I63.59, I63.6, I63.8, I63.9, I77.71 |
